# Intrapancreatic Fat and Risk of Pancreatic Ductal Adenocarcinoma—Pathophysiology, Clinical Implications, and Future Directions

**DOI:** 10.3390/ijms27187991

**Published:** 2026-09-08

**Authors:** Svenja Meyhöfer, Paula Lünswilken, Dimitris Grammatopoulos, Harpal Randeva, Jens U. Marquardt, Hendrik Lehnert

**Affiliations:** 1 Department of Internal Medicine 1, University of Lübeck, 23538 Lübeck, Germany; svenja.meyhoefer@uksh.de (S.M.); paula.luenswilken@uksh.de (P.L.); jens.marquardt@uksh.de (J.U.M.); 2 German Center for Diabetes Research (DZD e.V.), 85764 Neuherberg, Germany; 3 Warwick Medical School, University of Warwick, Coventry CV4 7AL, UK; d.grammatopoulos@warwick.ac.uk (D.G.); harpal.randeva@warwick.ac.uk (H.R.); 4 University Hospitals of Coventry and Warwickshire (UHCW), Coventry CV2 2DX, UK; 5 Römerwallklinik, 55131 Mainz, Germany

**Keywords:** pancreatic fat, obesity, cancer, GLP-1 receptor agonists, metabolic risk, carcinogenesis

## Abstract

Pancreatic adenocarcinoma (PDAC) remains one of the most lethal malignancies worldwide, with limited improvements in long-term survival despite significant progress in systemic therapy. This review focuses predominantly on pancreatic ductal adenocarcinoma (PDAC), the most common and best-studied pancreatic malignancy, while referencing other pancreatic neoplasms only where directly relevant to intrapancreatic fat biology. Obesity has emerged as a major modifiable risk factor for PDAC. In addition, increasing attention has focused on ectopic fat depots, particularly intrapancreatic fat, as potential mediators linking metabolic disease to pancreatic carcinogenesis. This narrative review summarizes the classification and epidemiology of PDAC, the relationship between obesity and pancreatic cancer risk, the biology of intrapancreatic fat, as well as putative mechanisms by which intrapancreatic fat may promote pancreatic carcinogenesis. In addition, we will review current diagnostic approaches, therapeutic and preventive considerations, and key unanswered questions for future research on the relevance of intrapancreatic fat deposits. Collectively, available evidence supports intrapancreatic fat as a biologically plausible and potential mediator of pancreatic cancer risk, warranting further prospective investigation.

## 1. Literature Search Strategy

This narrative review of the literature was conducted using PubMed, Scopus, and Web of Science databases. Search terms included ‘intrapancreatic fat’, ‘pancreatic steatosis’, ‘fatty pancreas’, ‘pancreatic ductal adenocarcinoma’, ‘pancreatic cancer risk’, ‘PDFF’, ‘obesity’, ‘GLP-1 receptor agonists’, ‘bariatric surgery’, and related terms. Studies published up to April 2026 were considered, with emphasis on original research articles, systematic reviews, and meta-analyses. No formal inclusion or exclusion criteria were applied given the narrative design. As this is a narrative rather than a systematic review, no formal quality-appraisal framework (e.g., SANRA) was applied to the included literature; this is acknowledged as a limitation in Section 8. Reference lists of key articles were additionally screened for relevant publications. The literature search was last updated in August 2026; titles and abstracts were screened for relevance by the authors, and full texts of potentially eligible articles were then reviewed before inclusion.

## 2. Classification and Epidemiology of Pancreatic Cancer

Pancreatic cancer is the seventh leading cause of cancer-related death worldwide and is projected to become the second leading cause in several high-income countries within the next decade [1,2,3,4]. The overwhelming majority of cases (>90%) are pancreatic ductal adenocarcinoma (PDAC), arising from the exocrine pancreas. Less common entities include pancreatic neuroendocrine neoplasms, acinar cell carcinoma, and solid pseudopapillary tumors.

PDAC is characterized by late presentation, aggressive biology, and a profound treatment-resistant phenotype in most patients already detected at the time of diagnosis. Five-year survival remains below 15%, largely due to advanced stage at diagnosis. Established risk factors include increasing age, smoking, chronic pancreatitis, type 2 diabetes, hereditary cancer syndromes, and obesity [3,4].

The clinical burden of PDAC is further aggravated by the lack of sensitive early detection strategies applicable to the general population. Most tumors remain asymptomatic until local invasion or metastatic dissemination has occurred, and current surveillance is largely restricted to individuals with hereditary syndromes or strong familial risk. This creates a major need for additional biomarkers that are biologically plausible, measurable at scale, and able to identify metabolically vulnerable individuals before irreversible neoplastic progression has occurred [1,3].

An important unresolved question is whether intrapancreatic fat (IPF) represents a true causal driver of pancreatic carcinogenesis or whether it may solely reflect an early consequence of subclinical pancreatic injury as well as metabolic dysbalances. Early neoplastic lesions may induce ductal obstruction and acinar cell loss, potentially leading to secondary fatty replacement of pancreatic tissue. This possibility complicates the interpretation of retrospective imaging studies and highlights the need for longitudinal investigations to establish temporal relationships [5].

This distinction is clinically important because pancreatic fat may represent both a marker and mediator of pancreatic vulnerability. On one hand, IPFD may reflect obesity-associated metabolic stress, aging, diabetes, or prior inflammatory injury. On the other hand, adipocyte infiltration and lipid accumulation within the pancreatic microenvironment may actively promote inflammation, fibrosis, epithelial injury, and stromal remodeling. Thus, IPFD should not be interpreted as a uniform imaging phenotype but rather as a biologically heterogeneous condition whose relevance may depend on anatomical distribution, cellular compartment, and coexistence of metabolic or inflammatory stressors [5].

## 3. Obesity as a Principal Risk Factor for Pancreatic Cancer

**Figure 1:**
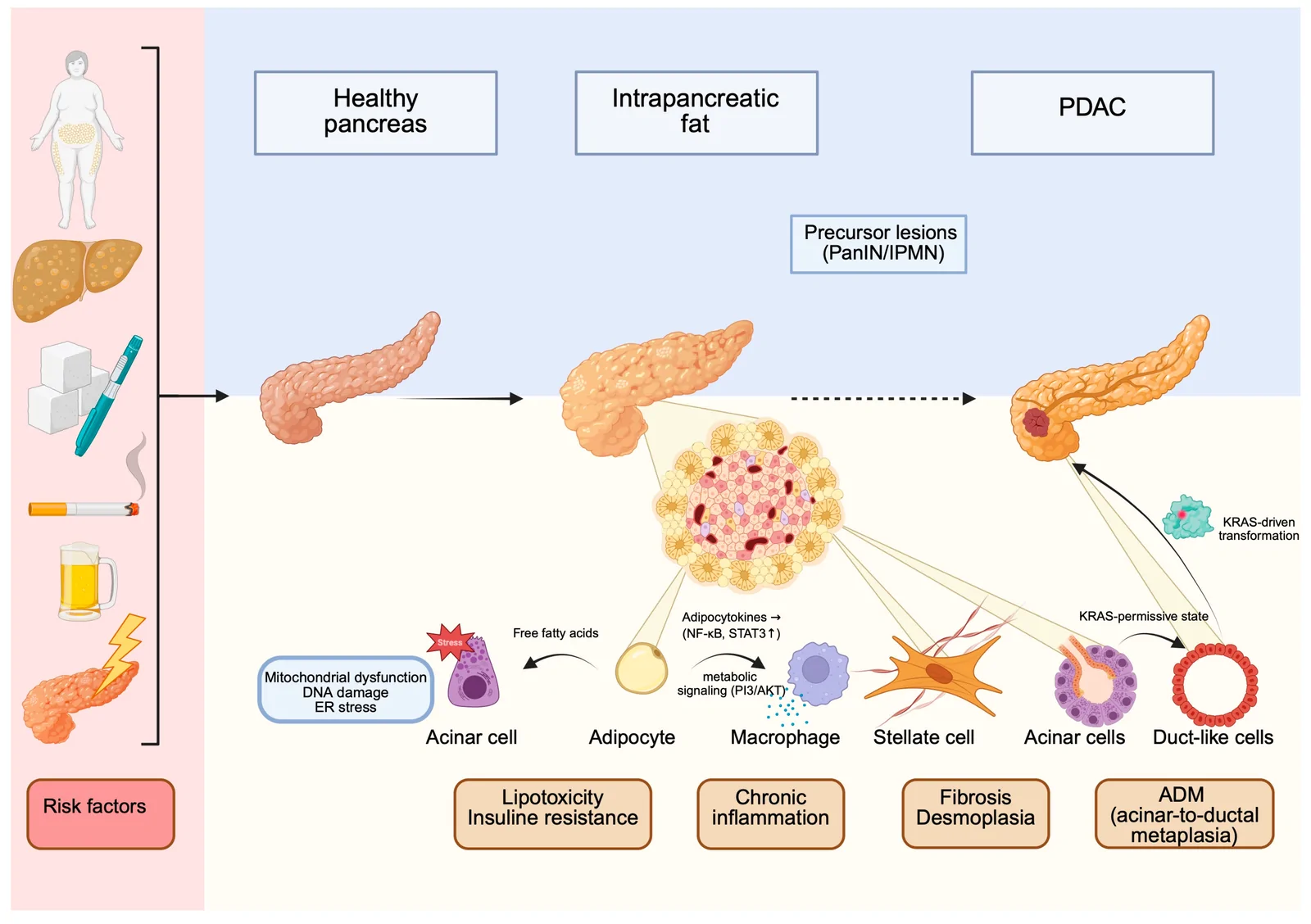
Proposed mechanisms linking intrapancreatic fat deposition to pancreatic ductal adenocarcinoma (PDAC). Obesity-related and lifestyle-associated metabolic risk factors such as visceral adipose tissue (VAT), metabolic dysfunction-associated steatotic liver disease (MASLD), diabetes mellitus, smoking, alcohol consumption and chronic pancreatitis contribute to intrapancreatic fat (IPF). Excessive pancreatic fat accumulation promotes lipotoxicity, insulin resistance, and release of free fatty acids and adipocytokines, resulting in mitochondrial dysfunction, endoplasmic reticulum stress, oxidative DNA damage, and chronic inflammation. Activation of inflammatory and metabolic signaling pathways, including NF-κB, STAT3, and PI3K/AKT, promotes macrophage recruitment, stellate cell activation, fibrosis, and desmoplastic remodeling. These processes may contribute to chronic pancreatitis and a pro-tumorigenic microenvironment permissive to acinar-to-ductal metaplasia (ADM) and oncogenic KRAS-driven transformation. Progressive neoplastic changes can subsequently lead to precursor lesions such as pancreatic intraepithelial neoplasia (PanIN) and intraductal papillary mucinous neoplasms (IPMN), ultimately promoting the development of PDAC.

Obesity is consistently associated with an increased risk of PDAC (Figure 1). Large prospective cohort studies and meta-analyses indicate that each 5 kg/m^2^ increase in body mass index (BMI) is associated with an approximately 10–15% increase in pancreatic cancer risk [6]. Central adiposity and increased visceral adipose tissue (VAT) appear to confer greater risk than BMI alone [7].

Mechanistically, obesity is associated with systemic insulin resistance (IR), hyperinsulinemia, chronic low-grade inflammation, and altered adipokine profiles, all of which have been implicated in pancreatic tumor initiation and progression [8,9,10]. Importantly, obesity promotes ectopic fat deposition in non-adipose organs, including the liver, skeletal muscle, and pancreas, raising the possibility that local fat accumulation may exert organ-specific protumorigenic effects that extend beyond the influence of generalized adiposity alone [11].

The relationship between obesity and pancreatic cancer is therefore unlikely to be fully explained by BMI alone. BMI does not distinguish between subcutaneous and visceral adiposity, nor does it capture ectopic lipid accumulation within organs. Visceral adipose tissue is metabolically active and contributes to systemic insulin resistance, inflammatory cytokine release, and altered adipokine signaling. These systemic effects may converge with local pancreatic fat accumulation to create a permissive endocrine–paracrine environment for early neoplastic change [7,8].

In support of this concept, a UK Biobank prospective cohort study of 42,599 participants found a 2-fold increased risk of pancreatic cancer associated with the presence of pancreatic steatosis and, importantly, also independently linked intrapancreatic fat deposits to acute pancreatitis and diabetes, demonstrating a scalable increase in risk alongside increasing pancreatic fat content [12]. These data support the concept that pancreatic steatosis may be clinically meaningful beyond its association with generalized obesity. Importantly, the simultaneous association of IPFD with diabetes, pancreatitis, and pancreatic cancer suggests that fatty pancreas may represent a shared upstream phenotype linking metabolic dysfunction, inflammatory pancreatic injury, and malignant transformation. This is particularly relevant because both diabetes and pancreatitis are themselves recognized risk factors for PDAC, making it plausible that IPFD may participate in a self-reinforcing disease network rather than acting as an isolated risk marker [5,12].

## 4. Biology of Intrapancreatic Fat

IPF represents a heterogeneous process involving both adipocyte infiltration and lipid accumulation within pancreatic parenchyma [13]. Histopathological studies distinguish intralobular fat, characterized by lipid droplets within acinar cells and islets, from extralobular (interlobular or peri-lobular) fat, consisting of mature adipocytes infiltrating the pancreatic interstitium [14,15]. These morphologically distinct compartments may exert different biological effects and should not be considered equally important.

The terminology surrounding IPFD remains inconsistent, which has contributed to conceptual and methodological ambiguity. “Fatty pancreas” is often used broadly in clinical imaging, whereas “intrapancreatic fat deposition” more specifically refers to measurable fat within the pancreatic parenchyma. Histologically, however, fat accumulation may include replacement of lost acinar tissue by adipocytes, interstitial adipocyte infiltration, or intracellular lipid accumulation in residual pancreatic cells. These processes may have different causes and consequences and should ideally be distinguished in future mechanistic and imaging studies [5,13,14]. For consistency, this review uses “intrapancreatic fat (IPF)” and “intrapancreatic fat deposition (IPFD)” as umbrella terms for excess pancreatic lipid encompassing both intralobular and extralobular fat, and uses “fatty pancreas” and “pancreatic steatosis” as synonymous imaging/histology descriptors of the same phenomenon rather than as mechanistically distinct entities, except where a specific histological subtype is explicitly named.

Extralobular fat has been linked to the presence of pancreatic intraepithelial neoplasia (PanIN), suggesting a spatially defined role in early carcinogenesis [15]. Cellular studies indicate that intrapancreatic adipocytes exert substantial endocrine and paracrine function, secreting adipokines and pro-inflammatory cytokines, thereby shaping a chronic pro-inflammatory microenvironment [16,17,18]. This environment also promotes oxidative stress, acinar cell injury, and activation of pancreatic stellate cells, key drivers of fibrosis and desmoplasia.

Macrophage infiltration is another prominent feature of IPF. Interestingly, the coexistence of inflammatory signaling and fibrosis mirrors key features of the tumor microenvironment in PDAC [16,17]. Metabolically, excess intrapancreatic fat promotes lipotoxicity through the release of free fatty acids and bioactive lipid species, inducing endoplasmic reticulum stress, mitochondrial dysfunction, and DNA damage in adjacent epithelial cells [19]. Emerging evidence further suggests that local crosstalk between adipocytes, immune cells, and pancreatic epithelial cells may promote a protumorigenic niche that precedes neoplastic transformation [20]. The inflammatory consequences of pancreatic fat may also depend on adipocyte size, local hypoxia, and immune-cell recruitment. Similar to visceral adipose tissue, enlarged adipocytes within or adjacent to pancreatic tissue may become dysfunctional, releasing chemokines and cytokines that attract macrophages and reinforce local inflammation. This creates a plausible mechanistic bridge between metabolic stress and epithelial injury, particularly in individuals with obesity, insulin resistance, or recurrent subclinical pancreatic inflammation [16,17,18] (Figure 1).

## 5. Mechanisms Linking Intrapancreatic Fat to Pancreatic Carcinogenesis

Pancreatic carcinogenesis is increasingly understood as a multistep process requiring both genetic alterations—most notably oncogenic KRAS mutations—and permissive microenvironmental conditions [21]. Experimental models demonstrate that KRAS activation alone is insufficient for malignant transformation without additional molecular alterations induced by accompanying inflammatory or tissue-damaging stimuli [22,23]. This model is consistent with the broader concept that oncogenic KRAS requires cooperation from non-genetic tissue injury signals. Chronic pancreatitis, acinar injury, and inflammatory remodeling can accelerate acinar-to-ductal metaplasia and PanIN formation in experimental systems. IPFD may act within this framework by lowering the threshold for injury-associated reprogramming and by maintaining a cytokine-rich microenvironment that facilitates persistence and expansion of preneoplastic clones [21,23].

IPF has been associated with several permissive pro-oncogenic signals, although causal evidence in humans remains limited. Adipocyte-derived cytokines promote sustained chronic inflammation and directly activate oncogenic signaling pathways, including NF-κB, STAT3, PI3K/AKT, and MAPK, ultimately promoting acinar-to-ductal metaplasia [16,18]. IPF has also been linked to pancreatic fibrogenesis through stellate cell activation, resulting in a desmoplastic microenvironment that further induces tumor progression. Adipocyte-rich pancreatic tissue may also alter local energy substrate availability. Free fatty acids released from intrapancreatic adipocytes can serve as metabolic substrates for adjacent epithelial or tumor cells, while simultaneously inducing oxidative and endoplasmic reticulum stress. Such metabolic stress may increase epithelial vulnerability to DNA damage and may promote adaptive signaling pathways that favor survival, proliferation, and inflammatory remodeling [16,19].

In addition to these experimental findings, the presence of IPF is implicated in several pancreatic pathologies, including diabetes mellitus, acute pancreatitis, chronic pancreatitis, and PDAC, supporting the concept that pancreatic steatosis contributes to a broad spectrum of pancreatic disease [24]. In addition, observational studies in human populations/patients demonstrate a strong association between pancreatic fat volume and pancreatic cancer risk, with adjusted odds ratios ranging from approximately 4 to 6 for PDAC among individuals with marked fatty infiltration [25,26]. However, the reported magnitude of association varies considerably across studies depending on imaging methodology, fat quantification thresholds, and population characteristics, and some estimates should be interpreted with caution pending prospective validation. IPF is also linked to the presence of pre-malignant precursor lesions such as PanIN and intraductal papillary mucinous neoplasms (IPMN), supporting a role in early neoplastic progression [15]. Still, the biological interpretation of these associations requires caution. Pancreatic fat may accumulate secondarily in areas of acinar atrophy or ductal obstruction, and small occult neoplasms may themselves induce local parenchymal replacement. Therefore, spatial proximity between fat and precursor lesions does not by itself prove causality (see Section 8 for a detailed discussion of causal inference and reverse causation). Future studies combining longitudinal imaging, histology, and molecular profiling will be required to determine whether IPFD precedes PanIN/IPMN development or instead marks tissue regions already altered by early neoplastic or inflammatory processes [5,15,24].

## 6. Diagnostic Work-Up of Intrapancreatic Fat

Intrapancreatic fat can be assessed using computed tomography (CT), magnetic resonance imaging (MRI), and endoscopic ultrasound (EUS). Among these modalities, MRI-based techniques, particularly chemical-shift imaging and proton density fat fraction (PDFF), currently provide the most accurate noninvasive quantification of pancreatic fat content and are increasingly regarded as the reference imaging approach for research studies [27,28,29] (Table 1).

Compared with CT, MRI enables continuous quantitative assessment of pancreatic fat with excellent reproducibility and strong correlation with both phantom standards and histopathological fat measurements, supporting its validity as a noninvasive biomarker of IPFD [28,29]. Nevertheless, accurate quantification remains challenging because pancreatic fat is distributed heterogeneously throughout the gland, with substantial variation between the head, body, and tail. Consequently, conventional region-of-interest (ROI) measurements may underestimate or overestimate overall pancreatic fat burden depending on sampling location, thereby limiting comparability across studies [29,30,31].

To overcome these limitations, recent studies have introduced artificial intelligence-assisted whole-organ segmentation, which permits automated volumetric quantification of pancreatic fat and more comprehensively captures its heterogeneous spatial distribution than conventional ROI-based approaches [30]. Although these techniques are promising, standardized acquisition protocols, validated diagnostic thresholds, and external validation across imaging platforms are still lacking before widespread clinical implementation can be recommended [27,30,31].

An additional unresolved issue is which imaging-derived fat parameter is biologically most relevant for pancreatic carcinogenesis. If IPFD primarily promotes cancer through local paracrine interactions, regional fat accumulation adjacent to ductal epithelial structures may be more important than total pancreatic fat content. Conversely, if pancreatic steatosis predominantly reflects systemic metabolic dysfunction, whole-organ fat burden may provide the more clinically relevant biomarker for risk stratification. Current imaging studies have not yet adequately distinguished between these possibilities [30,31].

Despite increasing recognition of IPF as a potential imaging biomarker for metabolic changes of the pancreas, no universally accepted diagnostic thresholds have been established, and intrapancreatic fat has not yet been incorporated into PDAC risk stratification algorithms [27,33].

EUS, including EUS-SWE, has been investigated as a complementary technique for qualitative assessment; however, EUS-SWE primarily quantifies tissue stiffness, which reflects fibrosis rather than fat content per se, and this distinction should be borne in mind when interpreting elastography findings of pancreatic steatosis [34]. It enables real-time visualization of parenchymal echogenicity but remains limited by operator dependency, the absence of validated quantitative criteria, and the inability to reliably distinguish intracellular lipid accumulation from adipocyte infiltration of the interstitium. Although preliminary EUS-based classification systems have recently been proposed, prospective validation in larger cohorts is required before routine clinical implementation can be recommended [33].

The clinical role of EUS may nevertheless become important in selected high-risk cohorts, because it allows simultaneous evaluation of parenchymal texture, ductal abnormalities, cystic lesions, and suspicious focal masses. In principle, EUS-based assessment of fatty pancreas could complement MRI in individuals already undergoing endoscopic surveillance. However, without validated quantitative thresholds and reproducible grading systems, EUS should currently be viewed as exploratory rather than definitive for IPFD-based risk assessment [33,34].

More recently, artificial intelligence-assisted whole-organ fat segmentation on MRI has emerged as a promising strategy to overcome the sampling limitations of conventional region-of-interest methods. Automated volumetric analysis may improve reproducibility across imaging centers and better capture the heterogeneous spatial distribution of pancreatic fat; the study by Dong et al. identified sex- and age-specific IPF thresholds using deep learning MRI quantification [11]. Still, large-scale external validation studies remain lacking [30,32].

## 7. Therapeutic and Preventive Considerations

There are currently no therapies approved specifically to target intrapancreatic fat for pancreatic cancer prevention. The rationale for therapeutic approaches targeting IPF is biologically plausible, with data from a recent systematic review of 23 longitudinal cohort studies showing that excessive IPF was associated with a 3-fold higher risk of PDAC and estimating that 23% of pancreatic cancer cases could theoretically be avoided by eliminating excessive IPF; this population-attributable-fraction estimate assumes a causal relationship between IPF and PDAC and should therefore be interpreted with caution given the current predominance of observational evidence [36]. It should be emphasized that, at present, no interventional study has demonstrated that pharmacological or surgical reduction of intrapancreatic fat lowers the incidence of pancreatic intraepithelial neoplasia (PanIN), intraductal papillary mucinous neoplasm (IPMN), or PDAC; the therapeutic implications discussed below are therefore hypothesis-generating rather than evidence-based.

From a preventive perspective, IPFD is attractive because it is potentially modifiable, in particular through lifestyle interventions that have been shown to consistently reduce ectopic fat and to improve insulin sensitivity. Unlike age, inherited susceptibility, or established precursor lesions, pancreatic fat may respond to sustained weight loss and improvement in insulin sensitivity. This raises the possibility that IPFD could eventually function both as a risk biomarker and as a surrogate endpoint in metabolic prevention trials. However, this concept remains hypothetical until reductions in pancreatic fat are shown to translate into lower rates of pancreatic precursor lesions or PDAC [5,36].

Lifestyle interventions consistently reduce ectopic fat and improve insulin sensitivity. GLP-1 receptor agonists (GLP-1 RAs) have shown reductions in related ectopic fat depots (e.g., liver and epicardial fat) in imaging studies, with direct pancreatic-fat imaging data still limited [37,38].

Mechanistically, GLP-1 RAs are shown to reduce circulating free fatty acids and attenuate adipose-driven inflammation, potentially mitigating pro-tumorigenic signalling within the tumor microenvironment [39]. However, although epidemiological studies consistently confirmed that GLP-1 receptor agonist use is not associated with increased risk for PDAC, direct positive implications for obesity-related cancers remain elusive [40]. Thus, dedicated prospective studies with PDAC as a primary endpoint and sufficient follow-up duration are required before any chemoprevention claims can be substantiated.

The interpretation of incretin-based therapies in this context requires separation of safety and prevention questions. Available epidemiological data do not support an increased risk of pancreatic cancer with GLP-1 receptor agonists or dual agonists, which is reassuring for their use in obesity and type 2 diabetes. However, absence of harm does not establish cancer-preventive efficacy. Demonstrating prevention would require long follow-up, careful adjustment for weight loss, diabetes duration, pancreatitis, smoking, and baseline cancer risk, and ideally imaging-based monitoring of pancreatic fat dynamics [17,39,40].

Other pharmacologic approaches, including sodium–glucose cotransporter-2 (SGLT-2) inhibitors, have also shown beneficial effects in reducing pancreatic fat in small clinical studies [5,41,42]. Whether these interventions translate into reduced pancreatic cancer risk remains unknown [5,38]. SGLT-2 inhibitors may reduce pancreatic fat indirectly through weight reduction, improved glycemic control, reduced insulin demand, and favorable effects on ectopic lipid distribution. Current studies, however, are small and primarily metabolic rather than oncologic. Therefore, their relevance for pancreatic cancer prevention remains speculative, although they provide proof of principle that pharmacological modulation of pancreatic fat is possible in humans [41,42].

Bariatric surgery may represent another important intervention, as substantial postoperative reductions in ectopic fat depots have been observed after marked weight loss. An important experimental study demonstrated that Roux-en-Y Gastric Bypass Surgery (RYGB) prevents the occurrence of pancreatic cell acinar carcinoma (ACC)—a rare exocrine pancreatic neoplasm biologically and clinically distinct from PDAC—so this finding should be interpreted as proof-of-concept for a fat-lowering mechanism rather than direct evidence of PDAC risk reduction in male and female Ngn3-Tsc1−/− mice through suppression of mTORC1 signaling [43].

Whether these reductions in pancreatic fat contribute to the lower incidence of obesity-associated malignancies observed after metabolic surgery remains an important area for future investigation [44,45]. Bariatric surgery also provides an additional model to study the reversibility of IPFD because it produces profound and sustained metabolic changes. Reductions in ectopic fat after surgery may reflect improved insulin sensitivity, reduced lipotoxicity, altered inflammatory tone, and changes in gut–pancreas endocrine signaling. Whether pancreatic fat reduction itself mediates part of the observed reduction in obesity-associated cancer risk remains unresolved and would require studies linking pre- and postoperative pancreatic imaging to long-term oncologic outcomes [44,45]. A recent UK Biobank study involving 25,547 participants further found that higher IPF independently increased risk for 12 multi-systemic diseases, including metabolic, cardiovascular, digestive, musculoskeletal, ophthalmologic, renal, and neurobehavioral disorders. This study identified a 7.35% pancreatic fat threshold as potentially clinically useful, and used Mendelian randomization to support causal inference. This observation renders novel therapeutic approaches even more relevant [46].

## 8. Limitations of Current Evidence

The current evidence linking IPF to pancreatic carcinogenesis remains limited due to substantial methodological and biological uncertainties in the existing studies. Consequently, it remains unclear whether pancreatic fat accumulation directly promotes carcinogenesis or merely is a bystander of an underlying metabolic dysfunction that is a known and independent risk factor for PDAC. Nevertheless, recent Mendelian randomization analyses as well as large prospective cohort studies suggest that the relevance of IPF extends beyond metabolic dysfunction and may play a causal role in disease development [47].

A further limitation is reverse causation. Early pancreatic cancer can induce new-onset diabetes, weight loss, ductal obstruction, and parenchymal atrophy, all of which may influence pancreatic fat appearance on imaging. Retrospective studies performed close to the time of cancer diagnosis are therefore particularly vulnerable to bias. Prospective studies with long latency between fat measurement and cancer diagnosis are better suited to address this problem, but even these require careful exclusion of occult cancer at baseline [5,12,47].

Importantly, there is substantial heterogeneity in the definition and quantification of intrapancreatic fat across studies. Computed tomography, conventional MRI, and PDFF techniques differ considerably in sensitivity as well as specificity. In addition, a patchy distribution of pancreatic fat frequently observed in imaging may further aggravate interobserver variability. This lack of standardized imaging methodology limits comparability between studies and may partly explain conflicting results in the literature [48]. Standardization should include acquisition technique, segmentation strategy, anatomical subdivision, and reporting of fat fraction thresholds. Without such harmonization, studies may classify different biological phenotypes under the same label of fatty pancreas. This is particularly problematic when comparing CT attenuation, qualitative echogenicity, MRI-PDFF, and AI-derived whole-organ segmentation, because each method captures overlapping but not identical tissue properties [32,48].

Moreover, most observational studies incompletely adjust for major confounding factors such as visceral adiposity, type 2 diabetes, smoking, chronic pancreatitis, and age. Because these factors are independently associated with both pancreatic steatosis and pancreatic cancer risk, confounding overall remains a major concern [49]. Nevertheless, recent prospective cohort data with extensive adjustment for visceral adiposity, hepatic steatosis and metabolic comorbidities indicate that the association between IPF and pancreatic disease persists after adjustment, suggesting that IPF may contribute to pancreatic disease risk independent of generalized adiposity and associated metabolic dysfunction [12].

Residual confounding is especially relevant because IPFD is strongly linked to age, obesity, diabetes, visceral adiposity, and pancreatitis. These variables may act simultaneously as causes, mediators, and consequences of pancreatic fat accumulation. Conventional multivariable adjustment may therefore be insufficient to fully separate independent effects. Mendelian randomization and repeated-measure prospective imaging can help strengthen causal inference, but neither approach fully replaces mechanistic human tissue studies [24,47].

The biological distinction between intracellular lipid accumulation and adipocyte infiltration of the pancreatic interstitium has also not been consistently addressed. These two forms of fat deposition may have distinct pathophysiological consequences, yet many imaging studies cannot reliably differentiate both types [26].

Finally, prospective interventional data are lacking. Although weight loss interventions and incretin-based therapies may reduce pancreatic fat content, no study has yet demonstrated that reducing IPF lowers the incidence of pancreatic intraepithelial neoplasia or pancreatic ductal adenocarcinoma. Therefore, the clinical relevance of targeting pancreatic fat remains uncertain [49].

An additional limitation is the relatively small number of mechanistic human studies directly correlating imaging-derived pancreatic fat measurements with histopathological findings. Bridging this gap will be essential to validate imaging biomarkers and strengthen biological interpretation [35].

## 9. Outlook and Future Research Directions

Key unanswered questions include direct clinical and/or mechanistic evidence that intrapancreatic fat depositions causally contribute to pancreatic carcinogenesis rather than reflecting broader metabolic dysfunction, i.e., evidence for a reduction in cancer incidence, as well as the interaction with genetic factors that confer PDAC susceptibility. Future studies should prioritize standardized imaging protocols, prospective cohort designs, and integration of molecular tissue as well as serum profiling—ideally spatial analyses—to better define the biological significance of IPF. Particular attention should be placed on the interaction between IPF, obesity-related inflammation, and key oncogenic alterations such as KRAS.

Future research should also clarify whether IPFD has clinical value beyond established risk factors. For implementation, it will be necessary to determine whether adding pancreatic fat measurements improves prediction models based on age, family history, diabetes, smoking, pancreatitis, cystic lesions, and obesity-related parameters. A clinically useful biomarker would need to improve discrimination, be reproducible across centers, and define actionable thresholds for intensified surveillance or preventive intervention [5,12,36,46].

A second priority is the integration of imaging with histopathology. Correlating MRI-derived pancreatic fat maps with resection specimens, spatial transcriptomics, immune-cell profiling, and stromal characterization may help identify which fat compartments are biologically relevant. Such studies could distinguish metabolically inert fatty replacement from inflammatory adipocyte infiltration that actively contributes to epithelial remodeling and tumor-permissive stroma [16,30,35].

## 10. Conclusions

Emerging evidence positions intrapancreatic fat deposition (IPFD) as a biologically plausible link between obesity-related metabolic dysfunction and pancreatic carcinogenesis. Converging histopathological, experimental, epidemiological, and imaging studies support the concept that IPFD contributes to a chronic pro-inflammatory, lipotoxic, and fibrogenic microenvironment that may facilitate KRAS-driven neoplastic transformation and tumor progression. Although longitudinal cohort studies and Mendelian randomization analyses increasingly support a causal relationship, definitive proof remains lacking because most available human data are observational and are limited by heterogeneous imaging methodology, residual confounding, and uncertainty regarding the temporal sequence between fat accumulation and early neoplastic change.

At present, IPFD should therefore be regarded as an emerging risk-associated phenotype rather than an established indication for pancreatic cancer surveillance. Nevertheless, standardized quantitative assessment of pancreatic fat has considerable potential to improve risk stratification, particularly in individuals with obesity, type 2 diabetes, chronic pancreatitis, new-onset diabetes, or hereditary susceptibility, where metabolic dysfunction and pancreatic cancer risk converge. Future research should focus on harmonized imaging protocols, prospective longitudinal cohorts, integration of imaging with molecular and spatial tissue profiling, and interventional studies determining whether reduction of pancreatic fat translates into lower incidence of pancreatic precursor lesions or PDAC.

If these challenges can be addressed, IPFD may evolve from a metabolic imaging finding into a clinically actionable biomarker that enables personalized pancreatic cancer risk assessment, identifies individuals most likely to benefit from intensified surveillance, and ultimately serves as a target for preventive metabolic interventions.

## Figures and Tables

**Table 1:** Diagnostic modalities for assessment of intrapancreatic fat deposition (IPFD).

Modality	Principle	Strengths	Limitations	Current Clinical Role
**Computed tomography (CT)**	Quantification of pancreatic attenuation (Hounsfield Units) relative to reference tissues	Widely available; routinely performed during abdominal imaging; detects advanced fatty replacement	Limited sensitivity for mild steatosis; radiation exposure; attenuation influenced by scanner settings and contrast administration; poor quantitative reproducibility	Incidental detection of fatty pancreas; not recommended for dedicated IPFD quantification [27,28]
**Magnetic resonance imaging (MRI)**	Chemical-shift imaging and proton density fat fraction (PDFF) quantify tissue triglyceride content	Highest accuracy and reproducibility; excellent correlation with histology; no ionizing radiation; quantitative assessment	Expensive; limited availability; heterogeneous fat distribution complicates ROI selection; lack of standardized acquisition protocols and diagnostic thresholds	Current reference standard for non-invasive IPFD assessment in research; promising biomarker for longitudinal studies [27,28,29,30,31]
**Artificial intelligence-assisted MRI**	Automated whole-pancreas segmentation and volumetric fat quantification using deep learning	Minimizes sampling bias; improves reproducibility; captures heterogeneous fat distribution; enables large-scale population studies	Limited external validation; different algorithms produce variable results; not yet standardized for routine practice	Emerging research tool with potential future clinical implementation [12,30,32]
**Endoscopic ultrasound (EUS)**	Qualitative assessment of pancreatic echogenicity relative to adjacent organs	Excellent spatial resolution; simultaneous evaluation of pancreatic morphology, cysts and small lesions; can be combined with tissue sampling	Highly operator-dependent; lacks validated quantitative grading; difficult to distinguish intracellular lipid from adipocyte infiltration	Adjunctive modality in specialized centers; exploratory assessment of IPFD [33]
**EUS shear-wave elastography (EUS-SWE)**	Measurement of tissue stiffness during EUS	May provide complementary information on fibrosis accompanying pancreatic steatosis	Limited clinical experience; no validated cut-offs; stiffness reflects fibrosis rather than fat alone	Investigational technique requiring prospective validation [34]
**Histopathology (reference standard)**	Direct microscopic assessment of adipocyte infiltration and intracellular lipid accumulation	Differentiates interlobular adipocytes from intracellular lipid; permits correlation with inflammation, fibrosis and PanIN lesions	Invasive; available only after surgery or biopsy; unsuitable for screening or surveillance	Gold standard for mechanistic studies and imaging validation [14,15,35]

## Data Availability

No new data were created or analyzed in this study. Data sharing is not applicable to this article.

## Abbreviations

The following abbreviations are used in this manuscript:

AKT	Protein kinase B
BMI	Body mass index
CT	Computed tomography
EUS	Endoscopic ultrasound
GLP-1 RA	Glucagon-like peptide-1 receptor agonist
IPF	Intrapancreatic fat
IPMN	Intraductal papillary mucinous neoplasm
IR	Insulin resistance
KRAS	Kirsten rat sarcoma viral proto-oncogene
MAPK	Mitogen-activated protein kinase
MRI	Magnetic resonance imaging
NF-κB	Nuclear factor kappa-light-chain-enhancer of activated B cells
PanIN	Pancreatic intraepithelial neoplasia
PDAC	Pancreatic ductal adenocarcinoma
PDFF	Proton density fat fraction
PI3K	Phosphoinositide 3-kinase
SGLT-2	Sodium-glucose cotransporter-2
STAT3	Signal transducer and activator of transcription 3
VAT	Visceral adipose tissue
